# Targeted silencing of miRNA-132-3p expression rescues disuse osteopenia by promoting mesenchymal stem cell osteogenic differentiation and osteogenesis in mice

**DOI:** 10.1186/s13287-020-1581-6

**Published:** 2020-02-13

**Authors:** Zebing Hu, Lijun Zhang, Han Wang, Yixuan Wang, Yingjun Tan, Lei Dang, Ke Wang, Zhongyang Sun, Gaozhi Li, Xinsheng Cao, Shu Zhang, Fei Shi, Ge Zhang

**Affiliations:** 1 The Key Laboratory of Aerospace Medicine, Ministry of Education, Air Force Medical University, Xi’an, 710032 Shaanxi China; 2 Department of Orthopedics, Affiliated Hospital of Air Force Aviation Medicine Research Institute, Air Force Medical University, Beijing, 100089 China; 3 State Key Laboratory of Space Medicine Fundamentals and Application, China Astronaut Research and Training Center, Beijing, 100094 China; 4 Institute for Advancing Translational Medicine in Bone & Joint Diseases, School of Chinese Medicine, Hong Kong Baptist University, Hong Kong SAR, China; 5 Department of Orthopedics, No. 454 Hospital of PLA, Nanjing, 210002 China

**Keywords:** BMSCs, Osteogenic differentiation, miRNA, Disuse osteopenia, Targeted delivery

## Abstract

**Background:**

Skeletal unloading can induce severe disuse osteopenia that often occurs in spaceflight astronauts or in patients subjected to prolonged bed-rest or immobility. Previously, we revealed a mechano-sensitive factor, miRNA-132-3p, that is closely related to the osteoblast function. The aim of this study was to investigate whether miRNA-132-3p could be an effective target for treating disuse osteopenia.

**Methods:**

The 2D-clinostat device and the hindlimb-unloaded (HU) model were used to copy the mechanical unloading condition at the cellular and animal levels, respectively. Mimics or inhibitors of miRNA-132-3p were used to interfere with the expression of miRNA-132-3p in bone marrow-derived mesenchymal stem cells (BMSCs) in vitro for analyzing the effects on osteogenic differentiation. The special in vivo antagonists of miRNA-132-3p was delivered to the bone formation regions of HU mice for treating disuse osteopenia by a bone-targeted (AspSerSer)_6_-cationic liposome system. The bone mass, microstructure, and strength of the hindlimb bone tissue were analyzed for evaluating the therapeutic effect in vivo.

**Results:**

miRNA-132-3p expression was declined under normal conditions and increased under gravitational mechanical unloading conditions during osteogenic differentiation of BMSCs in vitro. The upregulation of miRNA-132-3p expression resulted in the inhibition of osteogenic differentiation, whereas the downregulation of miRNA-132-3p expression enhanced osteogenic differentiation. The inhibition of miRNA-132-3p expression was able to attenuate the negative effects of mechanical unloading on BMSC osteogenic differentiation. Most importantly, the targeted silencing of miRNA-132-3p expression in the bone tissues could effectively preserve bone mass, microstructure, and strength by promoting osteogenic differentiation and osteogenesis in HU mice.

**Conclusion:**

The overexpression of miRNA-132-3p induced by mechanical unloading is disadvantageous for BMSC osteogenic differentiation and osteogenesis. Targeted silencing of miRNA-132-3p expression presents a potential therapeutic target for the prevention and treatment of disuse osteoporosis.

## Introduction

Throughout life, the bones are constantly remodeled by means of two coordinated and synchronized processes, including osteoblast-driven bone formation and osteoclast-driven bone resorption. This remodeling helps the bones adapt to changing loads with the optimized morphological structure and is therefore sensitive to mechanical stimulation alterations [1]. Skeletal unloading can disrupt the physiological process of bone remodeling and can induce severe bone loss, especially in weight-bearing bones. This kind of bone loss, clinically termed disuse osteoporosis, is characterized by a reduction in bone mass and the deterioration of skeletal microarchitecture without a change in the bone mineral to collagen ratio [2]. It often occurs in spaceflight astronauts or in patients subjected to prolonged bed-rest or immobility [2, 3]. Unfortunately, many effective treatments available for primary osteoporosis are not as effective for disuse osteoporosis because of the different etiology, pathophysiology, and resultant pathology. Therefore, more attention should be paid to developing a well-targeted treatment based on the mechanobiological pathogenesis.

It is generally agreed that impaired osteogenic differentiation and osteogenesis are important factors in the development of disuse osteopenia. Osteoblasts originate from mesenchymal stem cells (MSCs), a kind of multipotent stromal cell that can also differentiate into other cell types such as chondrocytes, fibroblasts, adipocytes, or myoblasts [4]. In response to special stimuli, MSCs commit to the osteogenic lineage and gradually differentiate into mature osteoblasts. The osteogenesis process, e.g., proliferative expansion, matrix deposition, maturation, and mineralization, is completed during the intermediate stepwise differentiation of osteoprogenitors and preosteoblasts into mature osteoblasts [5, 6]. In this process, the osteoblast lineage cells are sensitive to mechanical stimulation. Early MSCs respond to mechanical cues and switch to osteogenic lineage differentiation more often than other cell types, such as adipocytes [7, 8]. Early osteoprogenitors can respond with the expansion of clonal proliferation and the enhancement of differentiation [9, 10]. MC3T3-E1, a widely used preosteoblast lineage cell, is further promoted to differentiate and mineralize by mechanical stimuli, as evidenced by the increase in special gene markers [11, 12]. In contrast, the absence of mechanical stimulation is able to inhibit the processes of MSC proliferation and osteogenic differentiation [13, 14], increases osteoblast sensitivity to apoptosis and regression [15, 16], and finally leads to a decreased rate of bone formation. Therefore, a treatment strategy aimed at rescuing the impaired osteogenic differentiation from the commitment of MSC to the maturation of osteoblasts is one of the most common endeavors for disuse osteopenia therapy.

The mechanisms by which the transcription or regulatory factors are involved in MSC differentiation towards the osteogenic lineage have been proposed in many studies [17]. Among those, miRNAs that remain well conserved in different species have recently been revealed as important regulators in the lineage commitment of MSC, osteogenic differentiation, and bone formation [18–20]. Furthermore, the involvement of miRNAs in the mechanotransduction pathways of osteogenic differentiation has also been studied [21–23]. Our previous studies showed that miRNA-132-3p was obviously increased in both bone tissue and osteoblast cells in gravitational mechanical unloading [24]. Further experiments confirmed that the overexpression of miRNA-132-3p was able to inhibit osteoblast differentiation and mineralization partly by repressing E1A-binding protein p300 (EP300) protein translation, which further resulted in the suppression of the activity and acetylation of Runt-related transcription factor 2 (RUNX2), a key activator for MSC differentiation towards osteoblast lineage cells [25]. Disturbing the overexpression of miRNA-132-3p can effectively attenuate the negative effects of gravitational mechanical unloading on in vitro osteoblast function. Thus, we hypothesize that silencing miRNA-132-3p expression in bone tissues may rescue the impaired osteogenic differentiation from the osteogenic lineage commitment of MSC to the maturation of osteoblasts; and this may enhance the osteogenesis process to treat the bone loss induced by mechanical unloading.

In this study, we found that the expression of miRNA-132-3p gradually declined during bone marrow-derived mesenchymal stem cell (BMSC) osteogenic differentiation. Gravitational mechanical unloading can upregulate the expression level of miRNA-132-3p in BMSCs. Then, gain- or loss-of-function experiments demonstrated that miRNA-132-p is a negative regulator in the process of BMSC osteogenic differentiation. Further studies have shown that the inhibition of miRNA-132-3p in BMSCs could effectively attenuate the negative effects of gravitational mechanical unloading on the osteogenic differentiation of BMSCs in vitro. Then, the special inhibitor of miRNA-132-3p, termed antagomir-132, was delivered to the bone formation regions of hindlimb-unloaded (HU) mice where BMSCs differentiate into osteogenic lineage cells and osteogenesis takes place. The bone mass, microstructure, and strength of the hindlimb bone tissue were obviously improved when miRNA-132-3p was silenced in HU mice. This study provides a promising protective or therapeutic approach for disuse osteopenia.

## Methods

### Primary BMSC isolation, culture, and osteogenic differentiation

Primary BMSCs were isolated as described previously [26]. Briefly, 6–8 weeks old C57BL/6j mice were sacrificed by cervical dislocation after anesthesia. Then, femurs and tibiae were dissected from the trunk of the body in a sterile environment. The muscle and connective tissue on the bones were removed clearly. The bone marrow cells were collected in Dulbecco’s modification of Eagle’s medium (DMEM, Gibco, USA) supplemented with 10% fetal bovine serum (FBS, Gibco) and incubated at 37 °C with 5% CO_2_ in a humidified chamber. According to the preferential attachment to tissue culture plastic, BMSCs were isolated and purified through frequent medium changing and diminished trypsinization time. When cells were passaged to the third generation, BMSCs were able to be induced to differentiate towards osteoblast lineage with differentiation medium containing 10% FBS, 50 μg/ml ascorbic acid, 10 mM sodium β–glycerophosphate, and 100 nM dexamethasone (Sigma-Aldrich, USA).

### Clinostat-based gravitational mechanical unloading

2D-clinostat (developed by China Astronaut Research and Training Center) is an effective tool for simulating a microgravity environment on the ground [27]. In this experiment, it was used to weaken the gravitational mechanical stimulation to cells. Cells grown on the coverslips were rotated uniformly around a horizontal axis. Thus, the gravitational mechanical unloading condition was achieved because there is a vector-averaged reduction in the apparent gravity acting on the cell while the vessel rotates 360°. Briefly, BMSCs were seed on the coverslips at a density of 1 × 10^5^ cells and were cultured with the normal growth medium. When cell confluence reached approximately 40~50%, the coverslips were placed into the holders of a chamber filled with normal growth medium and kept 12.5 mm away from the chamber’s rotational axis. It should be noted that the bubbles were fully removed from the chamber. Finally, the chambers were placed into a clinostat and rotated around a horizontal axis at 24 rpm. The clinostat was placed in an incubator at 37 °C. After exposing to clinorotation, the coverslips were further placed in the six-well plates and incubated with osteogenic medium for osteogenic differentiation.

### Experimental animal group

Six-month-old male C57BL/6j mice purchased from the Animal Center of Air Force Medical University were individually caged and acclimatized to standard conditions for several days. Then, 36 mice were randomly divided into 6 groups: (1) baseline group (BL): mice were euthanatized and sampled at the beginning of experiment; (2) control group (CON): mice were raised in normal condition during the experiment; (3) hindlimb unloading group (HU): mice were submitted to hindlimb unloading experiment; (4) Hindlimb unloading plus (AspSerSer)_6_-liposome injection group (HU + Mock): mice were injected with the (AspSerSer)_6_-liposome before HU; (5) Hindlimb unloading plus (AspSerSer)_6_-liposome-antagomir-NC injection group (HU + antagomir-NC): mice were injected with the (AspSerSer)_6_-liposome-antagomir-NC before HU; and (6) Hindlimb unloading plus (AspSerSer)_6_-liposome-antagomir-132 injection group (HU + antagomir-132): mice were injected with the (AspSerSer)_6_-liposome-antagomir-132 before HU.

### Hindlimb-unloaded model

The hindlimb-unloaded model was used to copy disused bone loss on the hindlimbs by a tail suspension. To build the HU model, the tail was tightening moderately bound with a strip of adhesive surgical tape. The tape was attached to a chain hanging from a pulley so as to keep the hindlimbs suspended at a ~ 30° angle between the body and the floor. This allowed mice to freely move and access to food and water. The hindlimbs were submitted to further detection after 3 weeks of tail suspension.

### Western blot analysis

The protein expression of osteoblast differentiation markers was determined by western blot analysis. Briefly, cells were lysed using M-PER mammalian protein extraction reagent containing a protease inhibitor (Thermo Fisher Scientific, USA). And protein concentrations were tested with Pierce® BCA Protein Assay Kit (Thermo Fisher Scientific) according to the manufacturer’s specifications. Then, the lysates were separated on an 8% SDS/PAGE. After electrophoretic transfer on to nitrocellulose membranes (Thermo Fisher Scientific) and blocking with 5% milk solution, blots were incubated overnight at 4 °C with primary antibodies including anti-Runx2 rabbit monoclonal antibody (1:2000, Epitomics, CA), anti-Sp7/Osterix rabbit polyclonal antibody (1:1000, Abcam, UK), and GAPDH Rabbit Polyclonal Antibody (1:5000, Proteintech, China). Then, they were incubated with a horseradish peroxidase-conjugated secondary antibody (1:5000, Jackson, USA). The protein bands were detected and visualized by the imaging system (Tanon 5500, China) after being incubated with the SuperSignal™ West Pico Plus Chemiluminescent Substrate (Thermo Fisher Scientific). Densitometry analyses of the western bands were performed using the ImageJ Imaging software.

### qRT-PCR analysis

Total RNAs were extracted from the cells or bone tissues with the RNAiso Plus Reagent (Takara, Japan) according to the manufacturer’s instructions. Before using for polymerase chain reaction (PCR), the quality of total RNA was assessed with the optical density 260 nm/280 nm. For mRNA quantification, the first-strand cDNA was synthesized using a PrimeScript® RT Master Mix reagent kit (Takara). Amplification and quantification of the cDNA were conducted using SYBR® Premix Ex Taq™ II (Takara) in the CFX96 real-time PCR detection instrument (BIO-RAD, USA). The primers were listed as follows: *Runx2* (GenBank Accession NM_053470): F-5′-CCA TAA CGG TCT TCA CAA ATC C-3′ and R-5′-GCG GGA CAC CTA CTC TCA TAC T-3′; *Osx* (NM_001037632): F-5′-CAG TAA TCT TCG TGC CAG ACC-3′ and R-5′-CTT CTT TGT GCC TCC TTT TCC-3′; *Alp* (NM_013059): F-5′-AGA TGG ACA AGT TCC CCT TTG-3′ and R-5′-ACA CAA GTA GGC AGT GGC AGT-3′; *Col1a1* (NM_007742): F-5′-GAC ATG TTC AGC TTT GTG GAC CTC-3′ and R-5′-GGG ACC CTT AGG CCA TTG TGT A-3′; *GAPDH* (NM_008084): F-5′-CAG TGC CAG CCT CGT CTC AT-3′ and R-5′-AGG GGC CAT CCA CAG TCT TC-3′. GAPDH was used as an internal control. For miRNA quantification, PrimeScript™ RT Master Mix reagent kit (Takara) was used again to synthesize the cDNA. The Bulge-Loop™ miRNA qRT-PCR system to detected miRNA-132-3p was designed and purchased (RiboBio Biotechnology, China). The subsequent real-time PCR detection was the same as that of mRNA detection described above. U6 small nuclear RNA was used as an internal control.

### Mimic and inhibitor of miRNA-132-3p synthesis and usage

To achieve the gain- or loss-of-function of miRNA-132-3p, the inhibitor including antimir-132 used for in vitro and antagomir-132 used for in vivo, and the mimic of miRNA-132-3p were designed and synthesized with chemical modification by RiboBio Biotechnology Co., Ltd. Briefly, the antimir-132 was chemically modified, single-stranded oligonucleotides which at least contains a key sequence complementary to the seed-targeting 8-mer oligonucleotides of miRNA-132-3p. And the antagomir-132 was 3′ cholesterol-conjugated, 2′-*o*-methyl-modified antisense oligonucleotides that are fully complementary to miRNA-132-3p [28]. The mimic was a small, chemically modified double-strand RNA that mimics the sequence and function of miRNA-132-3p. In each experiment, we delivered the antimir-132 and mimic of miRNA-132-3p using Lipofectamine 2000 reagent (Invitrogen, USA) according to the manufacturer’s instructions. The antagomir-132 or antagomir-NC were prepared as described previously [29]. Briefly, the lyophilized delivery system (1.5 mg/kg body weight) was rehydrated by adding 0.5 ml DEPC-treated water containing antagomir-132 or antagomir-NC (4 mg/kg body weight) and were incubated for 20 min at room temperature. The entrapment procedure was performed immediately before use and then sterilized by passing through a 0.22-μm sterile filter. Specifically, it is hard to deliver the drugs via tail vein injection due to the wrapped tail in hindlimb-unloaded experiment. So, a 3-day consecutive pre-injection before HU was adopted to keep a high concentration of antagomir-132 in bones.

### Alkaline phosphatase activity assay

To examine alkaline phosphatase activity, BMSCs were washed with PBS and then lysed with M-PER mammalian protein extraction reagent (Thermo Fisher Scientific) for 30 min, and finally centrifuged at 14,000 rpm for 15 min. The supernatants were collected to determine their alkaline phosphatase activities using the ALP assay kit (Jiancheng Biotechnology, China). Protein concentrations were measured using the Pierce® BCA Protein Assay Kit. ALP activity (IU/L) was defined as the release of 1 nmol p-nitrophenol per minute per microgram of total cellular protein.

### Alizarin red staining

Alizarin red is a sort of dye which can bind to calcium ions and form depositing red nodules. Here, Alizarin red staining was used to assess the calcium deposition in mineralizing extracellular matrix. Briefly, cells were rinsed with DPBS three times and fixed with 70% cold ethanol for 1 h. Then, remove the ethanol and rinse the fixed cellular layer three times with ddH_2_O. Cells were stained with 40 mM Alizarin red S solution (PH 4.2) (Sigma) for 10 min at room temperature with gentle agitation. After staining, cultures were washed with ddH_2_O five times and incubated with DPBS for 15 min at room temperature. Finally, the red nodules were observed with an inverted light microscope and taken a picture with a digital camera.

### Micro-CT analysis

The trabecula microstructure of the femur in each group was evaluated by micro-CT (Siemens, Germany) with a resolution of 10.44 μm/slice. Briefly, the femurs were firstly fixed with 4% (v/v) paraformaldehyde for 24 h before submitting to scanning. The basic parameters of scanning energy were set as voltage 80 kV, current 500 mA, and exposure time 800 ms/frame over a 360° rotation. The angle of increment around the sample was set to 0.5°. After scanning, the 2D images were submitted to a workstation and reconstructed into a 3D microstructure. The area in 3D microstructure, which is approximately 1.5 mm away from the proximal epiphyseal growth plate, extended 2.0 mm length towards the femoral head and covered all the cancellous bone that was selected as the region of interest (ROI). The detailed 3D indices in the defined ROI were analyzed including bone mineral density (BMD), ratio of bone volume to tissue volume (BV/TV), ratio of bone surface to bone volume (BS/BV), trabecular number (Tb.N), trabecular thickness (Tb.Th), trabecular separation (Tb.Sp), and trabecular pattern factor (TPF) [30]. The operator who performed the scan analysis was blinded to the treatment associated with the specimens.

### Calcein double labeling

The mice in each group were injected intraperitoneally with Calcein (5 mg/kg body weight) twice at 10 days and 3 days, respectively, before euthanasia. At the end of the experiment, the bone tissue was dissected and fixed with 70% ethanol for 5 days. Then, the samples were embedded in methyl methacrylate without decalcification. After the tissue was sliced, the fluorescence of calcein was observed and the distance between the middle of two fluorescein labels was measured by ImageJ software. Finally, mineral apposition rate (MAR, μm/day) and bone formation rate per bone surface (BFR/BS, μm^3^/μm^2^/day) were calculated to evaluate the bone formation.

### Analysis of biomechanical properties

Biomechanical properties of the femur were tested by the three-point bending test with an electromechanical material-testing machine (Bose, USA). The femoral samples were placed on a bracket with a span length of 8 mm. And a loading speed of 0.02 mm/s was set to exert on the anterior surface of the diaphyseal mid-part. Load and deformation data were recorded and sampled at 50 Hz. The load-deflection curves were used to calculate max load at failure (N), stiffness (slope of the load-deflection curve, representing the elastic deformation, N/mm), and elasticity modulus (Gpa).

### Statistical analysis

All statistical analyses were performed with the SPSS software. The data are expressed as the mean ± SD of at least three independent experiments in vitro and six independent experiments in vivo. Comparisons were performed using a two-tailed *t* test or one-way ANOVA for experiments with more than two subgroups. A *P* value of less than 0.05 was considered significant.

## Results

### miRNA-132-3p inhibits the osteogenic differentiation of BMSCs in vitro

**Fig. 1:**
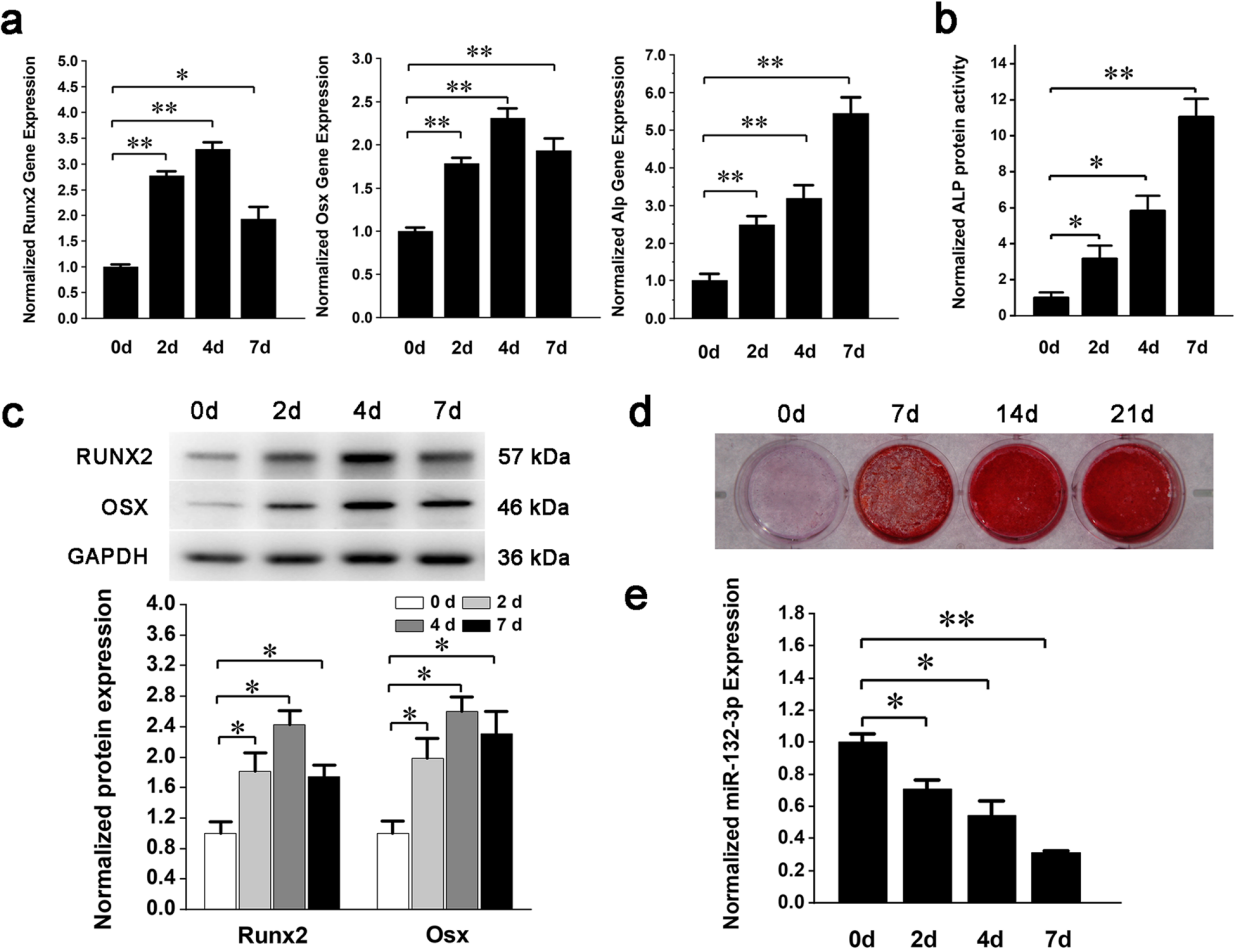
Osteogenic differentiation of BMSCs induced by an osteogenic medium. **a** Gene expression of osteogenic differentiation markers *Runx2*, *Osx*, and *Alp* was detected by qRT-PCR after osteogenic induction. **b** ALP protein activity was detected after osteogenic induction. **c** Protein expression of RUNX2 and OSX was detected by Western blot analysis and quantified using ImageJ software. **d** The calcific nodules of extracellular matrix were detected by Alizarin Red staining after 21 days of osteogenic induction. **e** Expression level of miRNA-132-3p was detected as BMSCs differentiated along the osteogenic lineage in vitro. Values are shown as mean ± SD, *n* = 3 in each group. *P < 0.05, **P < 0.01

**Fig. 2:**
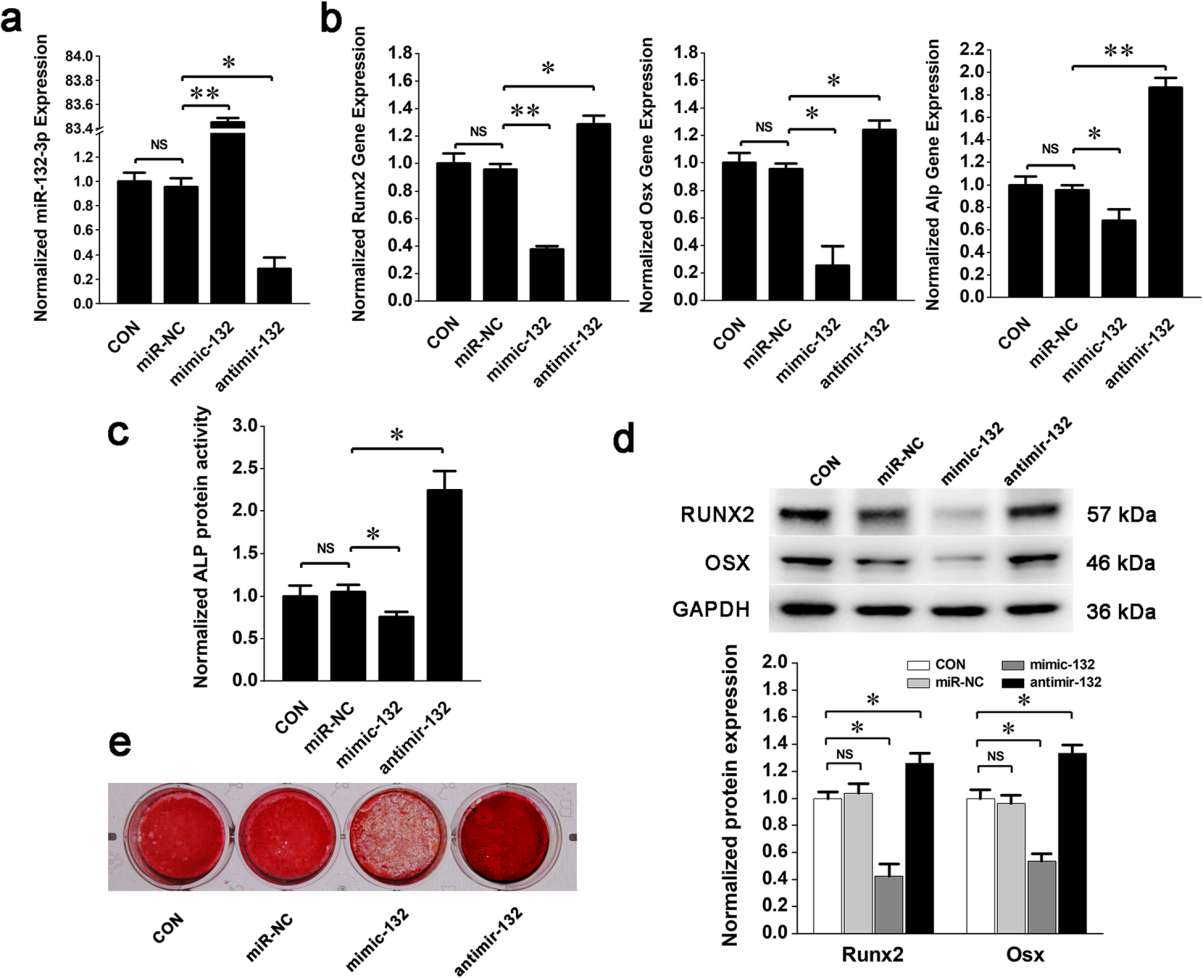
Function of miRNA-132-3p on osteogenic differentiation of BMSCs. BMSCs were transfected with the mimic (mimic-132), inhibitor (antimir-132), or their negative control (miR-NC) and were then induced to osteogenic lineage. **a** The expression of miRNA-132-3p after 4 days of osteogenic induction. **b** Gene expression of *Runx2*, *Osx*, and *Alp* after 4 days of osteogenic induction (compared to miR-NC). **c** The protein activity of ALP after 4 days of osteogenic induction. **d** Protein expression of RUNX2 and OSX after 4 days of osteogenic induction. **e** The calcific nodules of the extracellular matrix after 21 days of osteogenic induction. Values are shown as mean ± SD, *n* = 3. *P < 0.05, **P < 0.01. NS, no significant

To study the regulation of miRNA-132-3p on BMSC osteogenic differentiation, mouse primary BMSCs were identified and induced to differentiate towards the osteogenic lineage with the osteogenic medium. Differentiation was assessed by the expression levels of the specific transcription markers, *Runx2*, osterix *(Osx)*, and alkaline phosphatase *(Alp)* and by the mineralization of the extracellular matrix. The results showed that the expression of *Runx2*, *Osx*, and *Alp* (Fig. 1a), the enzyme activity of ALP (Fig. 1b), the protein expression of RUNX2 and OSX (Fig. 1c), and the mineralized nodules of the external matrix (Fig. 1d) were all significantly increased, which indicated that BMSCs were successfully induced to differentiate into osteoblast cells in vitro. In this process, miRNA-132-3p was detected but consequently declined (Fig. 1e). This shows a potential negative correlation with the osteogenic differentiation of BMSCs. Mimics or inhibitors were used to explore this potential correlation by intervening with the endogenic expression of miRNA-132-3p (Fig. 2a). The osteogenic differentiation phenotypes were decreased when miRNA-132-3p was upregulated, and the phenotypes were enhanced when miRNA-132-3p was downregulated in BMSCs (Fig. 2b–e). Thus, the conclusion can be drawn that miRNA-132-3p is a negative regulator in BMSC osteogenic differentiation.

### miRNA-132-3p mediates the unloading effects on BMSC osteogenic differentiation in vitro

**Fig. 3:**
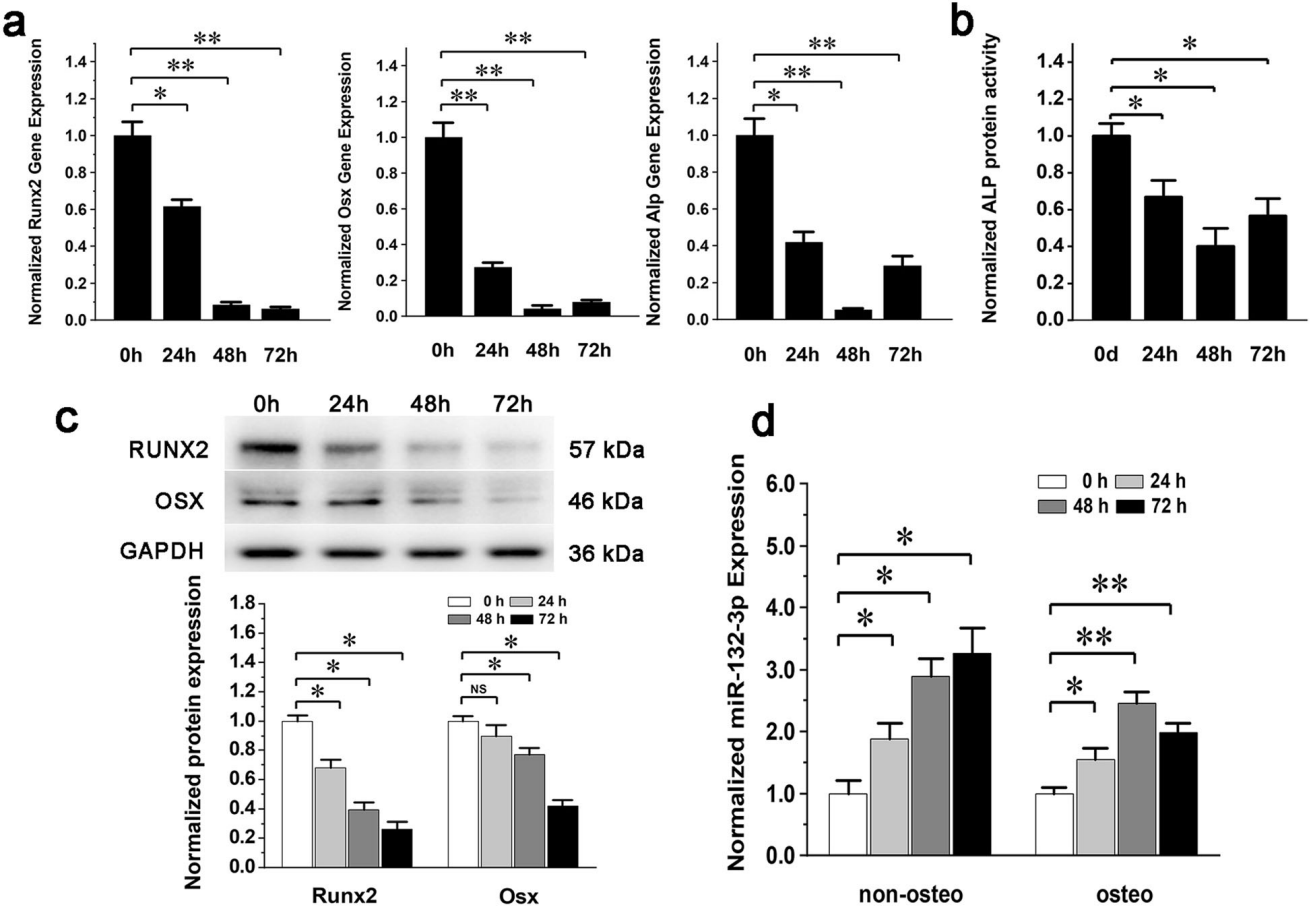
Effects of gravitational mechanical unloading on osteogenic differentiation of BMSCs. BMSCs were first exposed to a clinostat-based gravitational mechanical unloading environment for 0 h, 24 h, 48 h, 72 h and was then cultured with osteogenic medium for 4 days. **a** Gene expression of *Runx2*, *Osx*, and *Alp*. **b** Protein activity of ALP. **c** Protein expressions of RUNX2 and OSX. **d** The expression level of miRNA-132-3p in BMSCs after exposing to gravitational mechanical unloading (non-osteo, without osteogenic induction; osteo, further induced by an osteogenic medium). Values are shown as mean ± SD, *n* = 3. *P < 0.05, **P < 0.01. NS, no significant

**Fig. 4:**
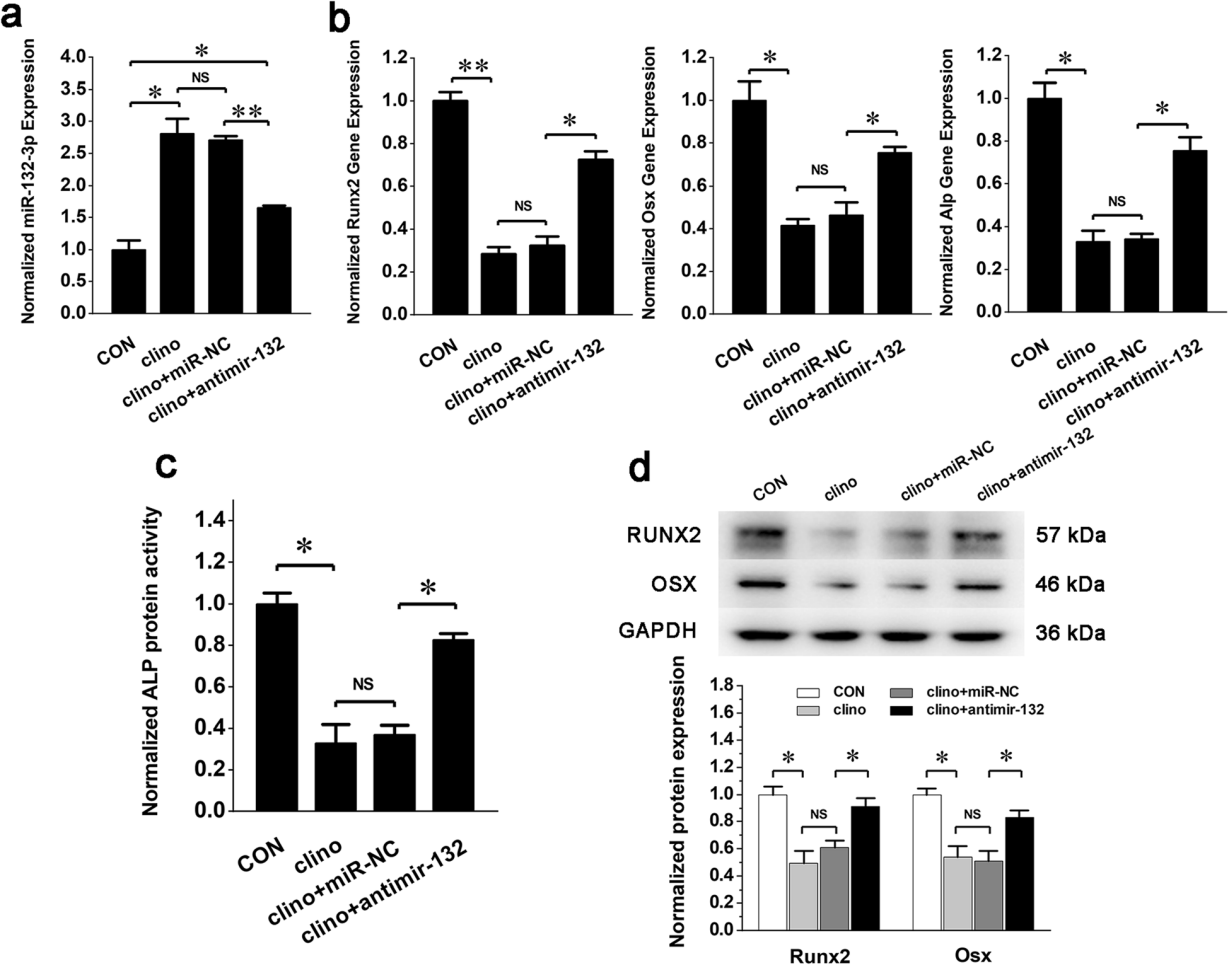
Downregulation of miRNA-132-3p partly attenuates the effects of mechanical unloading on BMSC osteogenic differentiation in vitro*.* BMSCs were transfected with the inhibitor of miRNA-132-3p for 6 h and were then exposed to gravitational unloading for 48 h. After the unloading exposure, BMSCs were cultured with osteogenic medium for 4 days. **a** miRNA-132-3p expression in BMSCs was analyzed after its antimiR was transfected. **b** Gene expression of *Runx2*, *Osx*, and *Alp*. **c** The protein activity of ALP was analyzed. **d** Protein expressions of RUNX2 and OSX were analyzed. Values are shown as mean ± SD, *n* = 3. *P < 0.05, **P < 0.01. NS, no significant

To verify whether miRNA-132-3p could respond to mechanical unloading in BMSC osteogenic differentiation, BMSCs were first exposed to a clinostat-based gravitational mechanical unloading environment and were then induced into the osteogenic lineage. The expression of *Runx2*, *Osx*, and *Alp* (Fig. 3a, c) and the enzymatic activity of ALP (Fig. 3b) were all gradually decreased, indicating that the osteogenic differentiation process of BMSCs was blocked by the unloading conditions. Meanwhile, the expression of miRNA-132-3p gradually increased as the exposure time prolonged (Fig. 3d), indicating that gravitational unloading can promote the expression of miRNA-132-3p during the aberrant osteogenic differentiation of BMSCs. Therefore, we hypothesized that miRNA-132-3p may be involved in this aberrant osteogenic differentiation. To verify this hypothesis, BMSCs were pretreated with the inhibitor of miRNA-132-3p and then submitted to the gravitational mechanical unloading experiments. The results showed that the silencing of miRNA-132-3p in BMSCs (Fig. 4a) could significantly promote the expression of osteogenic differentiation markers (Fig. 4b–d) and could effectively attenuate the negative effects of gravitational mechanical unloading on the osteogenic differentiation of BMSCs.

### Targeted delivery of antagomir-132 specifically decreases the miRNA-132-3p levels in the bones

**Fig. 5:**
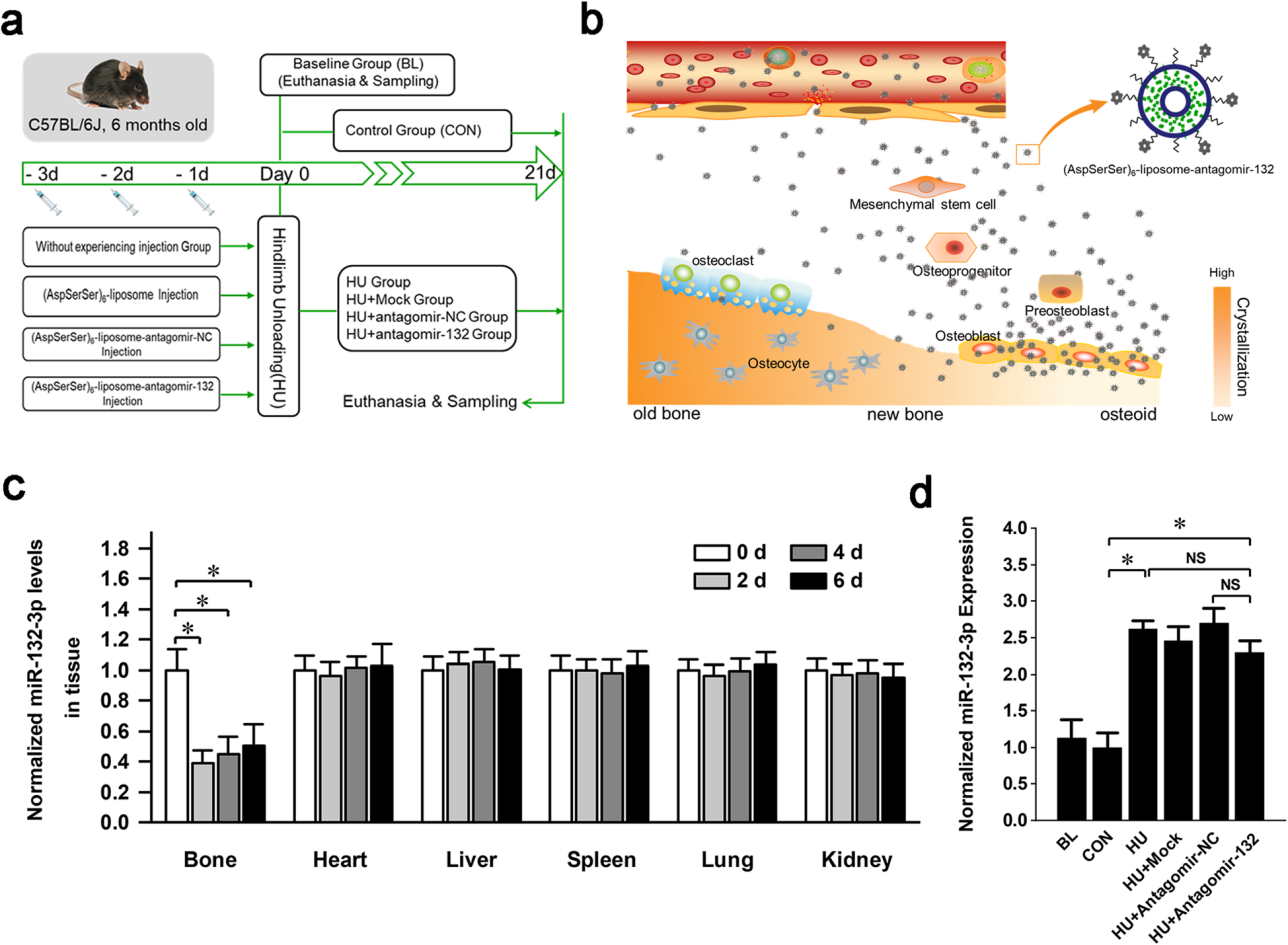
Targeted delivery of antagomir-132 specifically decreases miRNA-132-3p levels in bones. **a** A schematic diagram was used to illustrate the experimental design. **b** A schematic diagram was used to illustrate how antagomir-132 was selectively delivered to bone formation region. **c** Analysis of miRNA-132-3p expression in different tissues after a single injection of antagomir-132. **d** Analysis of miRNA-132-3p expression in the femur bone tissues of mice after hindlimb unloading for 21 days. Values are shown as mean ± SD, *n* = 6. *P < 0.05. NS, no significant

To obtain the in vivo specific silencing of miRNA-132-3p function in BMSC-derived osteogenic lineage cells of the mechanical unloading animal model, mice were subjected to hindlimb unloading for 21 days after 3 pulsed systemic administrations of antagomir-132 delivered by the bone-targeted (AspSerSer)_6_-cationic liposome system (Fig. 5a). According to the binding property of (AspSerSer)_6_-cationic liposome system, antagomir-132 would be mainly enriched in bone formation regions where various stages of osteogenic lineage cells reside (Fig. 5b). The mice were euthanized with one single injection of antagomir-132 for the miRNA-132-3p silencing specificity and efficiency test. Real-time PCR analysis showed that miRNA-132-3p expression in the bone tissues of experimental mice significantly decreased by approximately 60% 2 days after the injection and then slowly increased as antagomir-132 was exhausted in vivo, while no significant changes were observed in other non-skeletal organs, such as the heart, liver, lungs, and kidneys (Fig. 5c). At the end of the experiment, the expression levels of miRNA-132-3p in the mice of each group were detected (Fig. 5d). miRNA-132-3p was much higher in the HU group with or without the administration of antagomir-132 than it was in the baseline (BL) or control (CON) groups, indicating that mechanical unloading indeed triggered the overexpression of miRNA-132-3p, as we described previously. After the 21-day experiment, the level of miRNA-132-3p recovered to the HU level in the antagomir-132-treated group, possibly due to the quick response of miRNA-132-3p to mechanical unloading after the antagomir-132 were exhausted.

### Targeted silencing of miRNA-132-3p improves osteogenic differentiation and bone formation in vivo

**Fig. 6:**
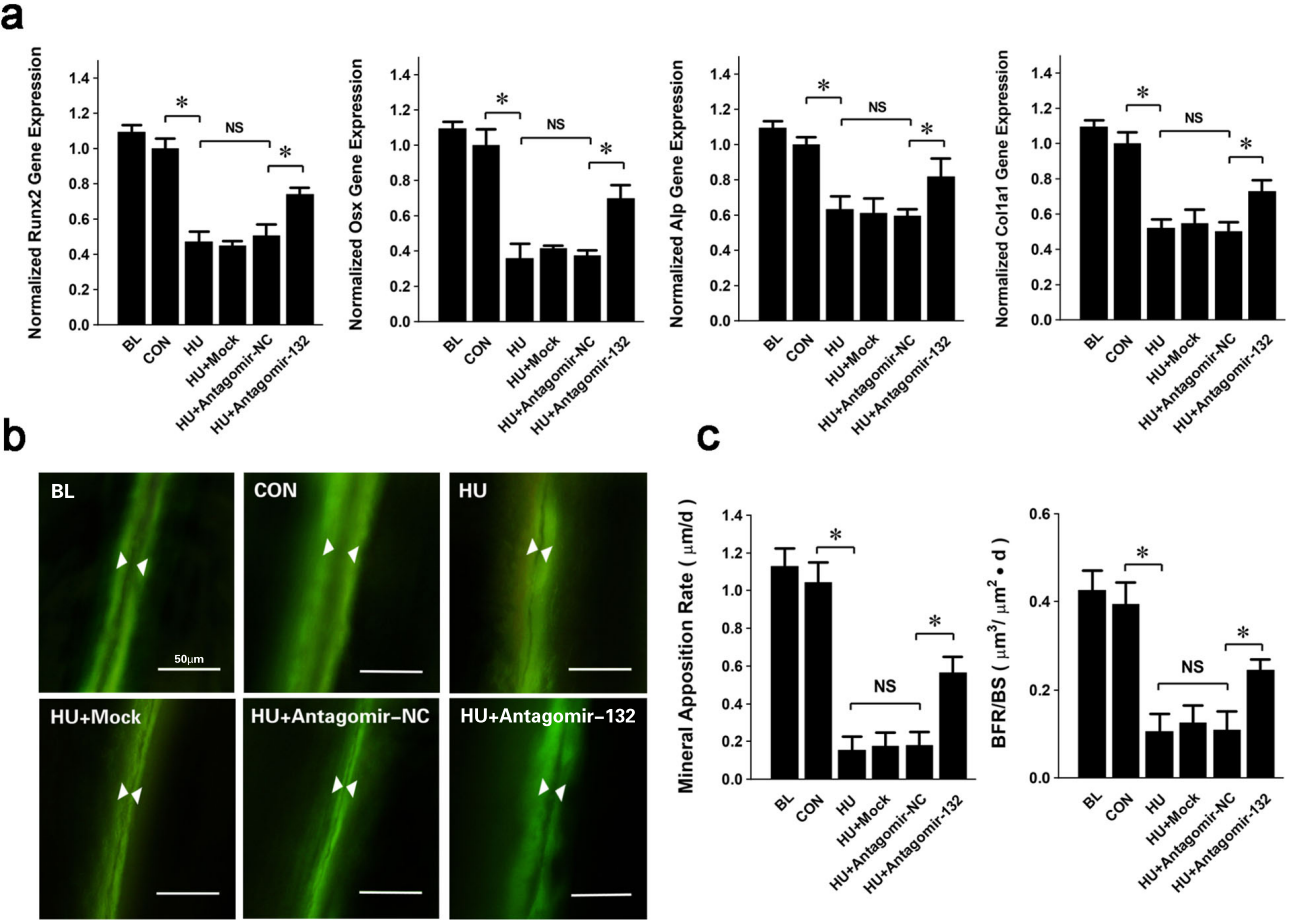
Targeted silencing of miRNA-132-3p improves osteogenic differentiation and osteogenesis in vivo. **a** The osteogenic differentiation phenotype was evaluated by determining the expression of *Runx2*, *Osx*, *Alp*, and *Col1a1*. **b** Fluorescent images of the calcein sediments in the region of bone formation. Scale bar = 50 μm. **c** Comparisons of mineral apposition rate (MAR) and bone formation rate per bone surface (BFR/BS) of the representative fluorescence images were calculated and analyzed. Values are shown as mean ± SD, *n* = 6. *P < 0.05. NS, no significant

To clarify the effect of silencing miRNA-132-3p expression on BMSC osteogenic differentiation in vivo and whether to rescue bone loss induced by mechanical unloading, osteogenesis was profiled after the injection of antagomir-132 in HU mice. Although the expression of the differentiation markers *Runx2*, *Osx*, *Alp*, and *collagen-1a (Col1a1)* did not recover to the normal levels of the CON group, they were all dramatically increased in the antagomir-132-treated group compared with those of the negative control (NC) group (Fig. 6a). This result indicated that the targeted silencing of miRNA-132-3p in the bone tissue effectively promoted the differentiation of osteogenic lineage cells. Thus, it can be further inferred that there may be more mature osteoblasts working and promoting extracellular matrix mineralization and new-bone formation. We performed a dynamic bone histomorphometric analysis of the distal femurs. The calcein double-labeling experiment showed a wider deposited line, which indicated that there was more new-bone formation during the same growth period (Fig. 6b). The mineral apposition rate (MAR) and bone formation rate per bone surface (BFR/BS) were significantly increased in the antagomir-132-treated group compared with those of the NC group (Fig. 6c). These results indicated that the targeting silencing of miRNA-132-3p expression in the bone tissues can improve osteogenic differentiation and bone formation in HU mice.

### Targeted silencing of miRNA-132-3p improves the microstructure and mechanical properties of the hindlimb

**Fig. 7:**
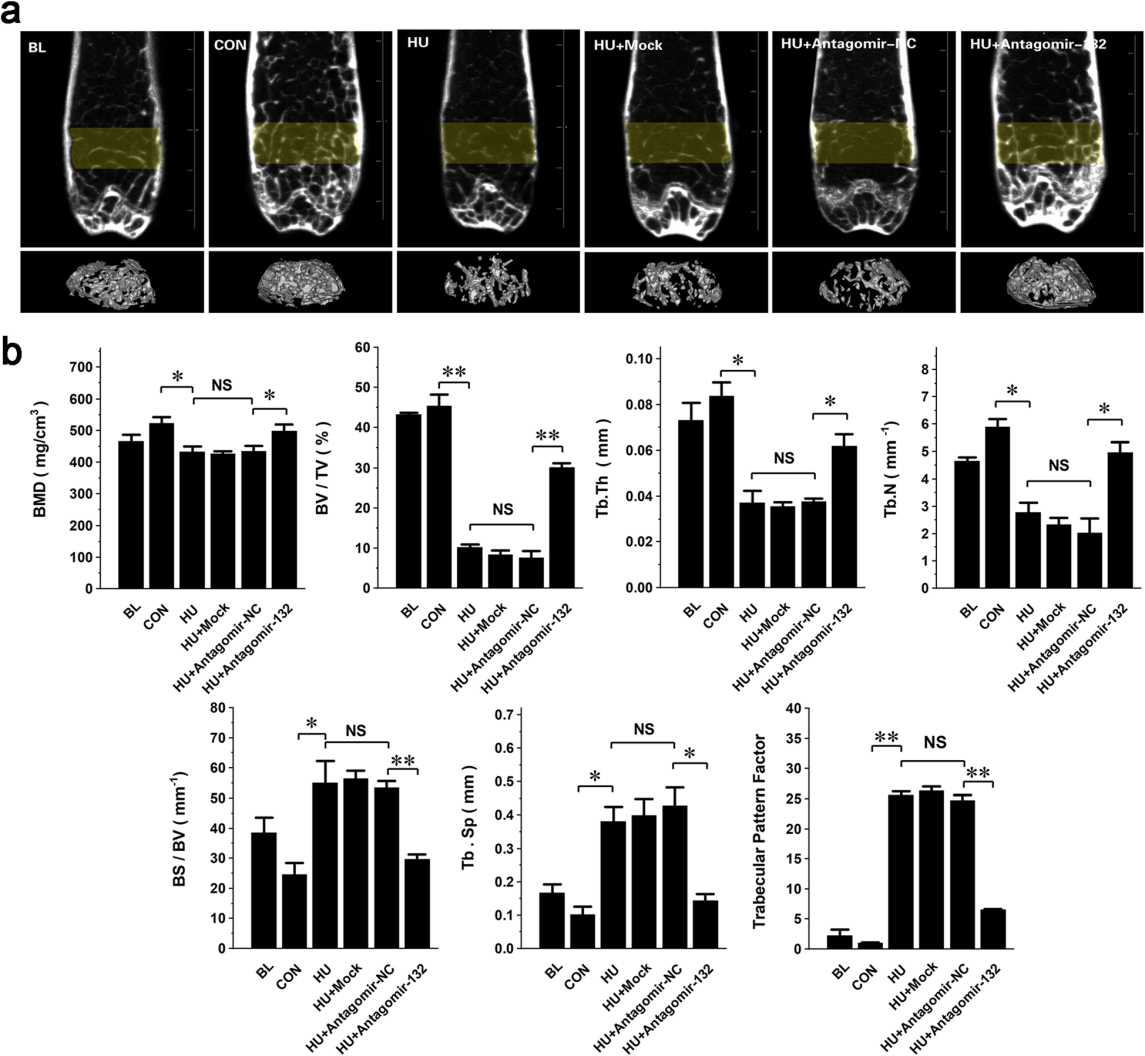
Targeted silencing of miRNA-132-3p improves the hindlimb bone microstructure of HU mice. **a** The region of interest (ROI) was selected as marked with yellow color. 3D reconstruction of ROI was shown at the bottom row of the images. **b** Three-dimensional microstructure parameters of the ROI, including bone mineral density (BMD), relative bone volume (BV/TV), trabecular thickness (Tb.Th), trabecular number (Tb.N), trabecular space (Tb.Sp), and trabecular pattern factor were analyzed. Values are shown as mean ± SD, *n* = 6. *P < 0.05. NS, no significant

**Fig. 8:**
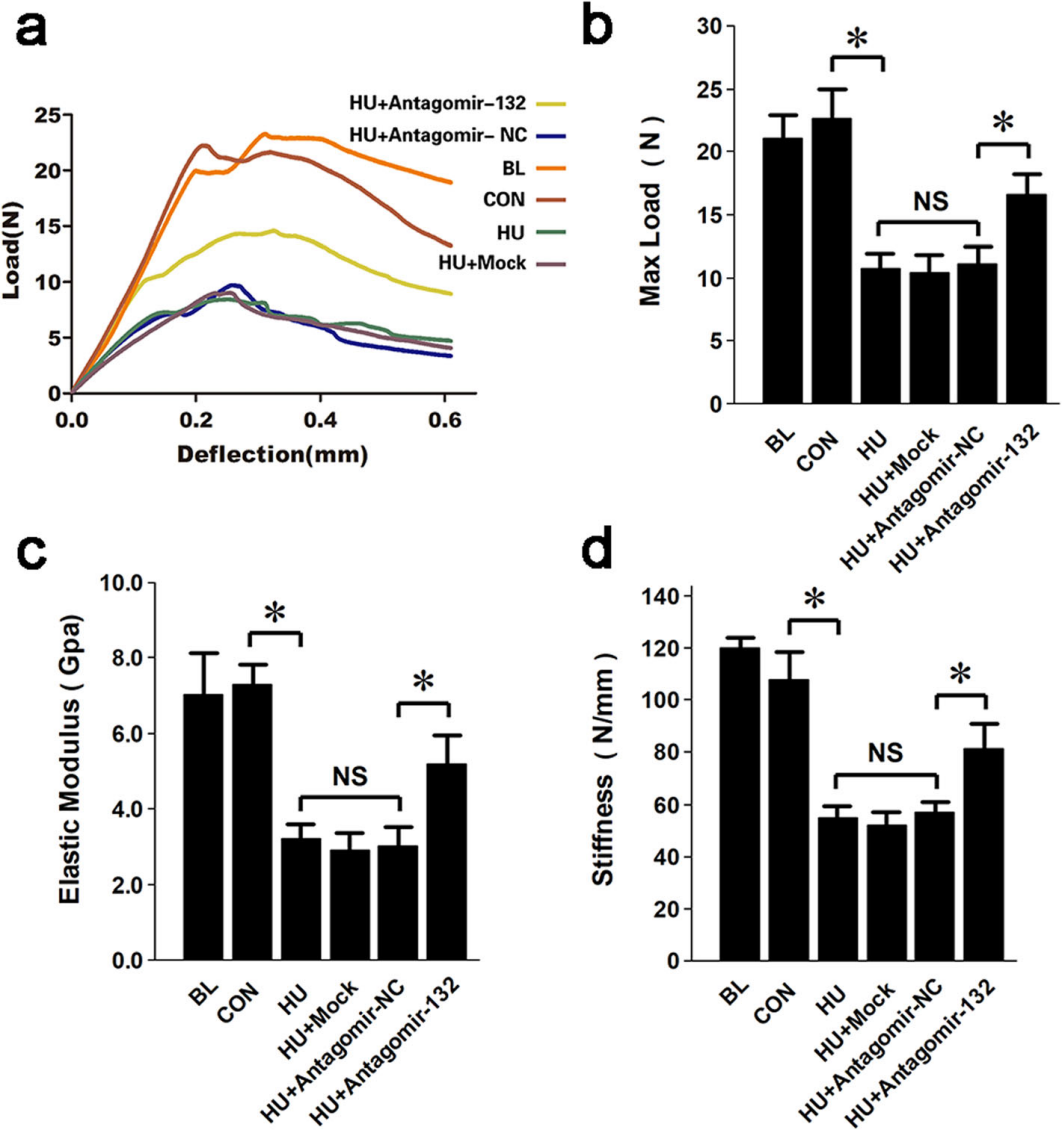
Targeted silencing of miRNA-132-3p enhances the hindlimb bone mechanical property of HU mice. Biomechanical properties of the femur were tested by the three-point bending test. **a** The load-deflection curves of the samples were drawn. **b** The biomechanical property parameters, including max load, elasticity modulus, and stiffness in each group were analyzed. Values are shown as mean ± SD, *n* = 6. *P < 0.05. NS, no significant

To observe the alteration of bone microstructure after the administration of antagomir-132, the femurs of each group were scanned by micro-CTs. The two- and three-dimensional reconstructed images of the bone displayed a sparse, fractured, and inconsecutive trabecular architecture in the HU, Mock, and NC groups and a relatively intact trabecular architecture in the antagomir-132-treated group (Fig. 7a). Three-dimensional architecture parameters showed that the bone mineral density (BMD), ratio of bone volume to total volume (BV/TV), trabecular thickness (Tb.Th), and trabecular number (Tb.N) were markedly increased while ratio of bone surface to bone volume (BS/BV), trabecular separation (Tb.Sp), and trabecular pattern factor (TPF) was markedly decreased in the antagomir-132-treated group compared with those of the HU, Mock, and NC groups (Fig. 7b). These data indicated that the targeted silencing of miRNA-132-3p was able to increase bone mass and restore the trabecular architecture of HU mice. Finally, the mechanical properties of the femurs in each group were evaluated by a three-point bend test. The load-deflection curves of samples were drawn (Fig. 8a). Three main biomechanical parameters, max load, stiffness, and elasticity modulus, were calculated and analyzed (Fig. 8b–d). The results showed that they were dramatically decreased in the HU, Mock, and NC groups compared with those of the CON and BL groups. However, the parameters were increased in the antagomir-132-treated group, although they did not recover to normal levels.

## Discussion

Currently, every effort is being made to look for the key factors responding to unloading at the cellular and molecular levels to develop effective and well-targeted treatments for disuse osteoporosis. Our previous study revealed a mechano-sensitive factor, miRNA-132-3p, which is upregulated in a gravitational mechanical unloading environment and can inhibit osteoblast differentiation and mineralization. In the present study, we demonstrated that the upregulated expression of miRNA-132-3p induced by gravitational mechanical unloading was able to inhibit the process of BMSC osteogenic differentiation. The targeted silencing of miRNA-132-3p in the bone formation region, where BMSCs differentiate into osteogenic lineage cells and osteogenesis takes place, can effectively preserve bone mass, microstructure, and strength by promoting osteogenic differentiation and osteogenesis in the hindlimb bone tissues of HU mice. This study presents a potential therapeutic target for the prevention and treatment of bone loss induced by unloading.

Previous studies have shown that miRNA-132-3p is involved in complicated and comprehensive regulatory networks, including neurological development [31], heart failure [32, 33], inflammation [34], angiogenesis [35], and even cancer [36]. However, the roles it plays in osteogenic differentiation, particularly during mechanical unloading conditions, are first reported by our group. As described previously, the overexpression of miRNA-132-3p induced by gravitational mechanical unloading can decrease the acetylation and transcriptional activity of RUNX2 by inhibiting the expression of the histone acetyltransferase EP300. *Runx2*, also known as core-binding factor a1 (*Cbfa1*), is the first transcription factor required for the determination of osteogenic lineage commitment and the activation of several key downstream proteins that maintain osteoblast differentiation and bone matrix [37]. This finding reminds us that the effects of miRNA-132-3p-mediated mechanical unloading on osteogenic differentiation may trace back to the osteogenic lineage commitment of MSCs and persist throughout the entire differentiation process.

Therefore, the expression and function of miRNA-132-3p were further studied in BMSC osteogenic differentiation processes. The results show that miRNA-132-3p expression declines as differentiation progresses under normal conditions. The extraneous upregulation of miRNA-132-3p can inhibit the expression of the osteogenic differentiation activator *Runx2* as well as its downstream markers *Osx* and *Alp*, while downregulation enhances their expression. A similar effect was observed on the mineralization of the extracellular matrix when the differentiation reached the end-stage. This indicates that the low-level expression of miRNA-132-3p is essential for the osteogenic differentiation of BMSCs, the maturation of osteoblasts and, consequently, osteogenesis. When the differentiation process is exposed to a gravitational mechanical unloading environment, the expression of miRNA-132-3p is increased, while BMSC osteogenic differentiation is decreased. Similarly, the inhibition of miRNA-132-3p expression is able to attenuate the negative effects of clinostat-based gravitational mechanical unloading on BMSC osteogenic differentiation. These results provide fundamental support for the therapeutic inhibition of miRNA-132-3p in vivo to rescue disuse osteopenia by recovering the impaired osteogenic differentiation and osteogenesis.

miRNAs have many advantages as therapeutic modalities, and several miRNAs are being tested in preclinical and clinical studies [38–40]. The miRNA sequences are short in length and are usually highly conserved across multiple vertebrate species [41]; for example, the mature sequences of miRNA-132-3p are completely consistent in humans, rats, and mice according to the miRBase database [42]. These characteristics make it relatively easy to target and allow the use of the same miRNA-modulating compound in preclinical efficacy and safety studies as well as in clinical trials [43]. Of course, challenges also exist for miRNA-based therapeutic development. The main challenges are how to maintain the stability and efficiency of miRNA antagonists or mimics and how to prevent off-target effects. To solve these problems, antagomir-132, an antagonist of miRNA-132-3p, was chemically modified to improve its binding affinity, biostability, and pharmacokinetic properties [28, 44]. Then, a delivery system targeting bone formation regions, the (AspSerSer)_6_-liposome system, was chosen to encapsulate and carry antagomir-132 to avoid the off-target effects [29]. This bone-specific targeting delivery system consisted of two moieties, including the navigational (AspSerSer)_6_ oligopeptide and the cationic liposome. The cationic liposome moiety was used to encapsulate the antagomir-132 and then was linked to the (AspSerSer)_6_ moiety. The (AspSerSer)_6_ oligopeptides have a high binding affinity for low-crystallized hydroxyapatite that is found in the bone formation regions rather than for high-crystallized hydroxyapatite that is found in the bone resorption regions. It is extremely suitable for our purpose because it can specially deliver antagomir-132 to bone formation regions where BMSCs are differentiating into osteoblast lineage cells at various differentiation stages.

The hindlimb-unloaded model is a well-accepted method to replicate disuse osteopenia on the hindlimbs by removing their weight-bearing loads. It has been demonstrated that HU can lead to a reduced bone mass, degenerated bone microstructure, and weakened bone resistance [45] as well as impaired osteogenic differentiation potentials [46, 47]. Based on the HU model, we tested the therapeutic effects of antagomir-132 on disuse osteopenia. Our results show that the targeted silencing of miRNA-132-3p can partially recover the impaired osteogenic differentiation evidenced by the increased differentiation activator and markers. In other words, the activities of osteoblast lineage cells are enhanced and more mature osteoblasts are devoted to the osteogenesis process. This is also verified by the newly formed bones in the double calcein labeling experiment. Micro-CT scans and three-dimensional reconstructions show that the bone mass is obviously increased and that the bone microstructure is recovered in the treatment group. Of course, the biomechanical properties of the femurs are strengthened correspondingly. These data demonstrate that the targeted silencing of miRNA-132-3p in the bone tissues can effectively alleviate disuse osteopenia with a variety of in vivo effects.

There are some limitations that should be noted in our study. This study mainly focuses on the therapeutic effects of the targeted silencing of miRNA-132-3p expression on disuse osteopenia. Therefore, the regulatory mechanism is not involved, and even the target gene of miRNA-132-3p, *Ep300*, elucidated in our previous paper, is not restated. In addition, due to the limitation of experimental materials, the pharmacokinetics of antagomir-132 and the time-dependent variation in miRNA-132-3p expression after the administration of antagomir-132 were both not tested. However, these limitations do not affect the therapeutic trials and should be addressed in future studies.

## Conclusion

In conclusion, this study was the first to demonstrate that the overexpression of miRNA-132-3p induced by mechanical unloading is disadvantageous for BMSC osteogenic differentiation and osteogenesis. Additionally, the targeted silencing of miRNA-132-3p expression in the bone tissues can preserve bone mass, microstructure, and strength by promoting osteogenic differentiation and osteogenesis in HU mice. These data provide new references on pharmaceuticals and treatment protocols for preventing or reducing disuse osteoporosis.

## Data Availability

The datasets supporting the conclusions of this article are included within the article.

## Abbreviations

ALP: Alkaline phosphatase
BFR/BS: Bone formation rate per bone surface
BL: Baseline
BMD: Bone mineral density
BMSCs: Bone marrow-derived mesenchymal stem cells
BS/BV: Ratio of bone surface to bone volume
BV/TV: Ratio of bone volume to tissue volume
Cbfa1: Core-binding factor a1
Clino: Clinorotation
Col1a1: Collagen, type I, alpha 1
CON: Control
DMEM: Dulbecco’s modification of Eagle’s medium
DPBS: Dulbecco’s phosphate-buffered saline
EP300: E1A-binding protein p300
FBS: Fetal bovine serum
GAPDH: Glyceraldehyde phosphate dehydrogenase
HU: Hindlimb-unloaded
MAR: Mineral apposition rate
miRNA: MicroRNA
MSC: Mesenchymal stem cell
NC: Negative control
OSX: Osterix
qRT-PCR: Quantitative real-time polymerase chain reaction
ROI: Region of interest
RUNX2: Runt-related transcription factor 2
Tb.N: Trabecular number
Tb.Sp: Trabecular separation
Tb.Th: Trabecular thickness
TPF: Trabecular pattern factor

## References

[1] Ruggiu A, Cancedda R (2015). Bone mechanobiology, gravity and tissue engineering: effects and insights. J Tissue Eng Regen Med.

[2] Lau RY, Guo X (2011). A review on current osteoporosis research: with special focus on disuse bone loss. J Osteoporos.

[3] Ohshima H (2010). Secondary osteoporosis UPDATE. Bone loss due to bed rest and human space flight study. Clin Calcium..

[4] Pittenger MF, Mackay AM, Beck SC (1999). Multilineage potential of adult human mesenchymal stem cells. Science..

[5] Ohata Y, Ozono K (2014). Bone and stem cells. The mechanism of osteogenic differentiation from mesenchymal stem cell. Clin Calcium.

[6] Javed A, Chen H, Ghori FY (2010). Genetic and transcriptional control of bone formation. Oral Maxillofac Surg Clin North Am.

[7] Benayahu D, Wiesenfeld Y, Sapir-Koren R. How is mechanobiology involved in mesenchymal stem cell differentiation toward the osteoblastic or adipogenic fate. J Cell Physiol. 2019;234(8):12133–41. 10.1002/jcp.28099 PMC13484546 30633367

[8] Luu YK, Pessin JE, Judex S, Rubin J, Rubin CT (2009). Mechanical signals as a non-invasive means to influence mesenchymal stem cell fate, promoting bone and suppressing the fat phenotype. Bonekey Osteovision.

[9] Liu C, Cabahug-Zuckerman P, Stubbs C, et al. Mechanical loading promotes the expansion of primitive osteoprogenitors and organizes matrix and vascular morphology in long bone defects. J Bone Miner Res. 2019;34(5):896–910. 10.1002/jbmr.3668 PMC8263903 30645780

[10] Brady RT, O’Brien FJ, Hoey DA (2015). Mechanically stimulated bone cells secrete paracrine factors that regulate osteoprogenitor recruitment, proliferation, and differentiation. Biochem Biophys Res Commun.

[11] Yan Y, Sun H, Gong Y (2016). Mechanical strain promotes osteoblastic differentiation through integrin-β1-mediated β-catenin signaling. Int J Mol Med.

[12] Zeng Z, Yin X, Zhang X, Jing D, Feng X (2015). Cyclic stretch enhances bone morphogenetic protein-2-induced osteoblastic differentiation through the inhibition of Hey1. Int J Mol Med.

[13] Yan M, Wang Y, Yang M (2015). The effects and mechanisms of clinorotation on proliferation and differentiation in bone marrow mesenchymal stem cells. Biochem Biophys Res Commun.

[14] Zayzafoon M, Gathings WE, McDonald JM (2004). Modeled microgravity inhibits osteogenic differentiation of human mesenchymal stem cells and increases adipogenesis. Endocrinology..

[15] Bucaro MA, Fertala J, Adams CS (2004). Bone cell survival in microgravity: evidence that modeled microgravity increases osteoblast sensitivity to apoptogens. Ann N Y Acad Sci.

[16] Gioia M, Michaletti A, Scimeca M (2018). Simulated microgravity induces a cellular regression of the mature phenotype in human primary osteoblasts. Cell Death Discov.

[17] Hu L, Yin C, Zhao F, Ali A, Ma J, Qian A. Mesenchymal stem cells: cell fate decision to osteoblast or adipocyte and application in osteoporosis treatment. Int J Mol Sci. 2018;19(2):​360. 10.3390/ijms19020360 PMC5855582 29370110

[18] Laine SK, Hentunen T, Laitala-Leinonen T (2012). Do microRNAs regulate bone marrow stem cell niche physiology. Gene..

[19] Dong S, Yang B, Guo H, Kang F (2012). MicroRNAs regulate osteogenesis and chondrogenesis. Biochem Biophys Res Commun.

[20] Lian JB, Stein GS, van Wijnen AJ (2012). MicroRNA control of bone formation and homeostasis. Nat Rev Endocrinol.

[21] Chang M, Lin H, Fu H, Wang B, Han G, Fan M (2017). MicroRNA-195-5p regulates osteogenic differentiation of periodontal ligament cells under mechanical loading. J Cell Physiol.

[22] Liu L, Liu M, Li R (2017). MicroRNA-503-5p inhibits stretch-induced osteogenic differentiation and bone formation. Cell Biol Int.

[23] Frith JE, Kusuma GD, Carthew J (2018). Mechanically-sensitive miRNAs bias human mesenchymal stem cell fate via mTOR signalling. Nat Commun.

[24] Hu Z, Wang Y, Sun Z (2015). miRNA-132-3p inhibits osteoblast differentiation by targeting Ep300 in simulated microgravity. Sci Rep.

[25] Ducy P, Zhang R, Geoffroy V, Ridall AL, Karsenty G (1997). Osf2/Cbfa1: a transcriptional activator of osteoblast differentiation. Cell..

[26] Soleimani M, Nadri S (2009). A protocol for isolation and culture of mesenchymal stem cells from mouse bone marrow. Nat Protoc.

[27] Patel MJ, Liu W, Sykes MC (2007). Identification of mechanosensitive genes in osteoblasts by comparative microarray studies using the rotating wall vessel and the random positioning machine. J Cell Biochem.

[28] Krützfeldt J, Rajewsky N, Braich R (2005). Silencing of microRNAs in vivo with ‘antagomirs’. Nature..

[29] Zhang G, Guo B, Wu H (2012). A delivery system targeting bone formation surfaces to facilitate RNAi-based anabolic therapy. Nat Med.

[30] Bouxsein ML, Boyd SK, Christiansen BA, Guldberg RE, Jepsen KJ, Müller R (2010). Guidelines for assessment of bone microstructure in rodents using micro-computed tomography. J Bone Miner Res.

[31] Miller BH, Zeier Z, Xi L (2012). MicroRNA-132 dysregulation in schizophrenia has implications for bothneurodevelopment and adult brain function. Proc Natl Acad Sci U S A.

[32] Liu X, Tong Z, Chen K, Hu X, Jin H, Hou M (2018). The role of miRNA-132 against apoptosis and oxidative stress in heart failure. Biomed Res Int.

[33] Ucar A, Gupta SK, Fiedler J (2012). The miRNA-212/132 family regulates both cardiac hypertrophy and cardiomyocyte autophagy. Nat Commun.

[34] Gutiérrez-Vázquez C, Rodríguez-Galán A, Fernández-Alfara M (2017). miRNA profiling during antigen-dependent T cell activation: a role for miR-132-3p. Sci Rep.

[35] Anand S, Majeti BK, Acevedo LM (2010). MicroRNA-132-mediated loss of p120RasGAP activates the endothelium to facilitate pathological angiogenesis. Nat Med.

[36] Gougelet A, Pissaloux D, Besse A (2011). Micro-RNA profiles in osteosarcoma as a predictive tool for ifosfamide response. Int J Cancer.

[37] Schroeder TM, Jensen ED, Westendorf JJ (2005). Runx2: a master organizer of gene transcription in developing and maturing osteoblasts. Birth Defects Res C Embryo Today.

[38] Bouchie A (2013). First microRNA mimic enters clinic. Nat Biotechnol.

[39] Janssen HL, Reesink HW, Lawitz EJ (2013). Treatment of HCV infection by targeting microRNA. N Engl J Med.

[40] Rottiers V, Obad S, Petri A (2013). Pharmacological inhibition of a microRNA family in nonhuman primates by a seed-targeting 8-mer antimiR. Sci Transl Med.

[41] Bartel DP (2004). MicroRNAs: genomics, biogenesis, mechanism, and function. Cell..

[42] Kozomara A, Griffiths-Jones S (2014). miRBase: annotating high confidence microRNAs using deep sequencing data. Nucleic Acids Res.

[43] van Rooij E, Kauppinen S (2014). Development of microRNA therapeutics is coming of age. EMBO Mol Med.

[44] Krützfeldt J, Kuwajima S, Braich R (2007). Specificity, duplex degradation and subcellular localization of antagomirs. Nucleic Acids Res.

[45] Peres-Ueno MJ, Stringhetta-Garcia CT, Castoldi RC (2017). Model of hindlimb unloading in adult female rats: characterizing bone physicochemical, microstructural, and biomechanical properties. PLoS One.

[46] Markina EA, Andrianova IV, Shtemberg AS, Buravkova LB (2018). Effect of 30-day hindlimb unloading and hypergravity on bone marrow stromal progenitors in C57Bl/6N mice. Bull Exp Biol Med.

[47] Pan Z, Yang J, Guo C (2008). Effects of hindlimb unloading on ex vivo growth and osteogenic/adipogenic potentials of bone marrow-derived mesenchymal stem cells in rats. Stem Cells Dev.

